# Japanese secular trends in birthweight and the prevalence of low birthweight infants during the last three decades: A population-based study

**DOI:** 10.1038/srep31396

**Published:** 2016-08-09

**Authors:** Yo Takemoto, Erika Ota, Daisuke Yoneoka, Rintaro Mori, Satoru Takeda

**Affiliations:** 1 Department of Obstetrics and Gynaecology, Juntendo University, Tokyo, Japan; 2 St. Luke’s international university, Graduate school of nursing science, Global Health Nursing, 10-1 Akashicho Chuo-ku Tokyo 104-0044, Japan; 3 Department of Statistical Science, School of Multidisciplinary Sciences, SOKENDAI (The Graduate University for Advanced Studies), Tokyo, Japan; 4 Department of Health Policy, National Center for Child Health and Development, Tokyo, Japan

## Abstract

Since low birthweight has been correlated with both neonatal and long-term health, we performed this epidemiological study to evaluate the Japanese secular trends in mean birthweight and the prevalence of preterm/term low birthweight infants during the last three decades. We used population-based birth certificate data from January 1979 to December 2010. Time trends were analysed using a linear regression model. During the study period, we observed a significant decrease in the mean birthweight for singleton live births (3,152 ± 436 g in 1979 and 3,018 ± 421 g in 2010 p < 0.001) and an increase in the prevalence of preterm/term low birthweight infants. A 96.3% increase in the proportion of term low birthweight infants was observed during the study period (2.7% in 1979 and 5.3% in 2010). In addition, an increased proportion of preterm/low birthweight infants born to younger women was observed (<35 years vs. ≥35 years). These trends may be related to changing patterns in Japanese women’s nutritional status and the relatively strict recommended limit on weight gain during pregnancy. Understanding the long-term trends for singleton births may allow us to identify the associated risk factors and reduce the future socioeconomic burden that is associated with low birthweight infants.

Several studies in developed countries have revealed increases in the mean birthweight and the proportion of high birthweight infants during recent decades^1^,^2^. In contrast, Japanese population-based data indicate that the mean birthweight has gradually declined from 3,200 g in 1979 to 3,020 g in 2009^3^. Similar results (decreasing mean birthweight and increasing prevalence of low birthweight [LBW] infants) were observed in a Japanese study that was performed using data collected between 1980 and 2000 from the Children and Infant Survey dataset, which has been collected every 10 years since 1950^4^. Mean birthweight significantly decreased from 3,189 ± 422 g in 1980 to 3,033 ± 429 g in 2000. Furthermore, the authors found that the prevalence of LBW infants increased from 4.2% in 1980 to 8.3% in 2000. These trends may have been related to increases in preterm deliveries and multiple gestations.

Birthweight is an important indicator of a newborn’s well-being, the intrauterine environment, and the mother’s nutritional status during pregnancy. In this context, the Developmental Origins of Health and Disease Theory states that a reduced birthweight is associated with both neonatal mortality and morbidity and also with long-term health conditions, such as cardiovascular disease, type 2 diabetes mellitus, hypertension, dyslipidaemia, and some cancers^5^,^6^,^7^. Thus, the recent increase in the prevalence of LBW infants may be associated with a significant socioeconomic burden in the future, despite the extremely low neonatal mortality rate in Japan^8^. Nevertheless, to the best of our knowledge, no studies have examined secular trends among LBW infants or evaluated their potential causes using population-based data for singleton live births in Japan. Moreover, it is important to understand the secular trends for singleton births, as the proportion of singleton births remains very high (98.8% in 1979 and 98.1% in 2010)^8^. Therefore, the present study aimed to assess Japanese trends in mean birthweight and the prevalence of term and preterm LBW infants during the last three decades and clarify risk factors, such as parity and maternal age.

## Results

**Figure 1:**
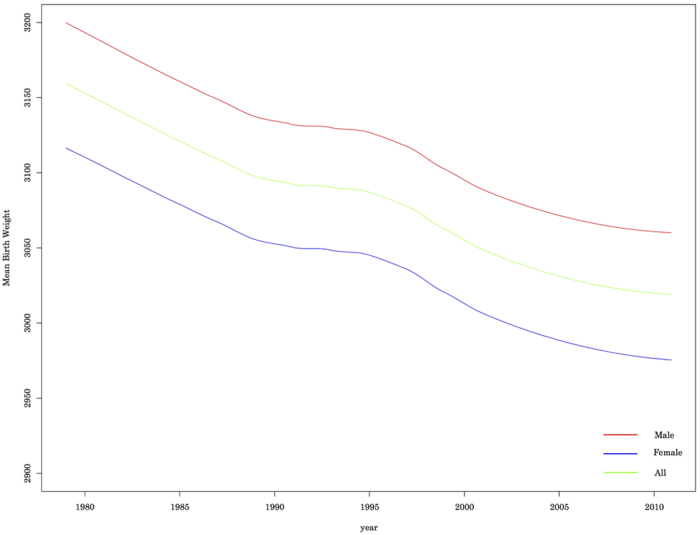
Secular trends in mean birthweight over 30 years.

Based on the Ministry of Health, Labour and Welfare data, we evaluated all singleton live births from 1979 to 2010 in Japan. Figure 1 shows the changes in mean birthweight, which decreased significantly from 3,152 ± 436 g in 1979 to 3,018 ± 421 g in 2010. The mean birthweight for male infants decreased from 3,193 ± 442 g in 1979 to 3,059 ± 427 g in 2010, and the mean birthweight for female infants decreased from 3,109 ± 426 g in 1979 to 2,974 ± 409 g in 2010 (Fig. 1 and Table 1).

**Figure 2:**
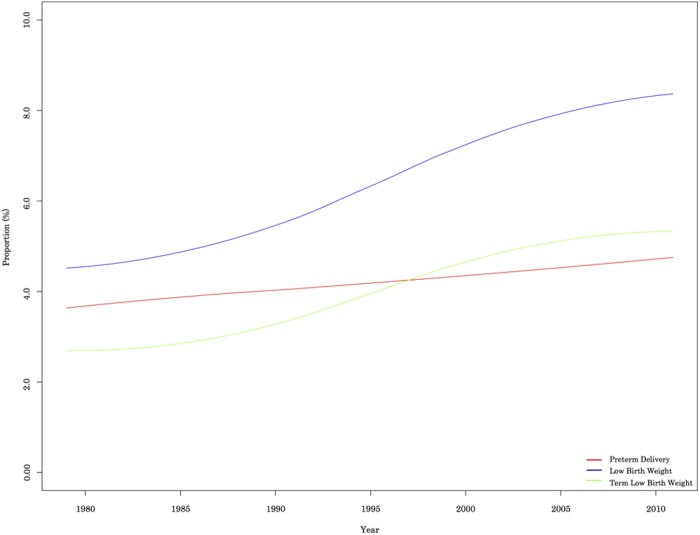
Secular trends in prevalence of preterm, LBW, and term LBW infants.

**Figure 3:**
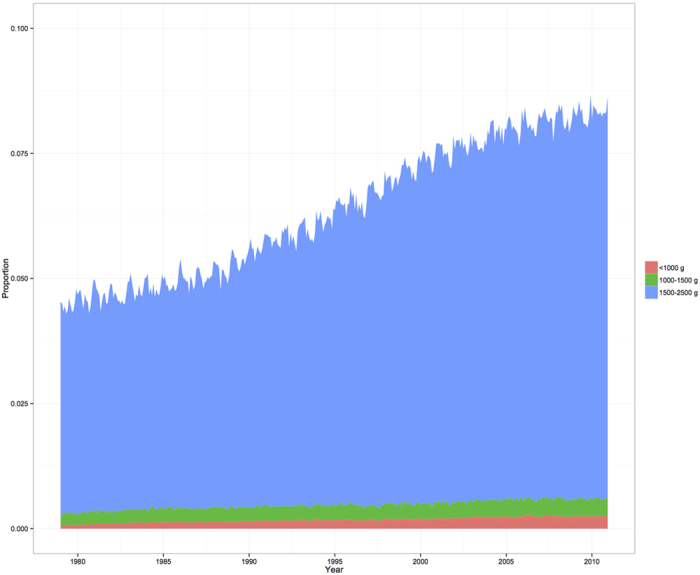
Secular trends in LBW infants according to their birthweight.

The prevalences of preterm, term, and LBW births are shown in Fig. 2. The prevalence of preterm births increased by 34%, from 3.5% in 1979 to 4.7% in 2010. This increase might have contributed to the increased prevalence of LBW infants, although the prevalences of LBW and term LBW infants exhibited much more dramatic increases during the study period. The prevalence of LBW infants increased from 4.5% in 1979 to 8.3% in 2010, and the prevalence of term LBW infants increased from 2.7% in 1979 to 5.3% in 2010 (Fig. 2 and Table 1). Figure 3 shows that the proportion of infants with a birthweight from 1,500–2,500 g exhibited the greatest proportional increase, compared to infants with very LBW (<1,500 g) or extremely LBW (<1,000 g).

Table 2 shows the prevalences of LBW infants according to maternal age. The prevalence of LBW infants exhibited a 91.5% increase among 25–34-year old women (from 4.14% in 1979 to 7.93% in 2010). A relatively smaller increase (27.8%) was observed in the prevalence of LBW infants among mothers ≥35 years old (from 7.44% in 1979 to 9.54% in 2010).

To evaluate the association between maternal age and the prevalence of preterm/LBW infants, the AORs for women ≥35 years old are shown in Table 3. After adjusting for infant sex and birthplace, we found that the odds ratios for preterm births among women ≥35 years old steadily decreased from 2.14 (95% CI: 2–2.28) to 1.50 (95% CI: 1.46–1.55) among nulliparous women and from 2.15 (95% CI: 2.07–2.22) to 1.30 (95% CI: 1.26–1.33) among multiparous women. Similar trends were observed in the AORs for LBW infants among women ≥35 years old, which significantly decreased from 2.06 (95% CI: 1.95–2.18) to 1.34 (95% CI: 1.31–1.37) among nulliparous women and from 1.90 (95% CI: 1.83–1.97) to 1.21 (95% CI: 1.18–1.24) among multiparous women (Table 3).

## Discussion

The present epidemiological study evaluated Japanese secular trends in birthweight and the prevalence of LBW infants using population-based data from 1979–2010. To the best of our knowledge, this is the first report regarding these trends among Japanese singleton live-birth infants. The present study revealed that the prevalence of term LBW infants (relatively mature, but <2,500 g) increased during the last three decades. Interestingly, the mean maternal height during this period increased significantly from 155.0 ± 5.1 cm in 1980 to 157.9 ± 5.3 cm in 2000, which has been accompanied by a Westernization of the Japanese lifestyle and eating habits^4^. Moreover, the prevalence of shorter mothers (<150 cm) decreased from 14.5% in 1980 to 4.6% in 2000^4^. In contrast, we found that the mean birthweight for singleton infants decreased, and the prevalence of term LBW infants increased, although other developed countries have experienced increases in mean birthweight^1^,^2^.

A previous Japanese study evaluated a smaller dataset from the Children and Infant Growth Survey (n = 11,746) and found that the proportion of LBW infants increased between 1980 and 2000. The authors also found that a low pre-pregnancy body mass index (BMI) and low maternal weight gain during pregnancy were risk factors for LBW^4^. Furthermore, the prevalence of underweight Japanese women (BMI of <18.5 kg/m^2^) who were of reproductive age increased significantly during the study period. Specifically, the prevalence of underweight among 20–29-year-old women increased from 13.1% in 1980 to 29.0% in 2010, and the prevalence among 30–39-year-old women increased from 7.9% in 1980 to 14.4% in 2010^9^. In some previous studies, maternal undernutrition was associated with increased risk of spontaneous preterm birth and LBW, though the mechanisms are still not clear^10^,^11^.

Low pre-pregnancy BMI and inadequate weight gain during pregnancy were associated with spontaneous preterm birth and foetal growth restriction. In underweight women, lower plasma volume results in a lower uteroplacental blood flow, which causes decreased transfer of nutrients to the foetus and a reduction in foetal growth^10^. In addition, maternal malnutrition is associated with deficiencies of multiple micronutrients. A previous review showed that decreased micronutrients correlated with increases in clinical infections, which could be a significant cause of spontaneous preterm birth^11^.

In Japan, pregnant women with BMIs from 18.5–25 kg/m^2^ are recommended to limit their weight gain to 7–12 kg during pregnancy^12^. In contrast, the recommended weight gain in the US is 11.3–15.9 kg for women in the same BMI group^13^. Moreover, the recommended weight gain is 9–12 kg for Japanese women with BMIs <18.5 kg/m^2^, compared to 12.7–18.1 kg for the same group in the US. Although these recommendations aim to achieve an appropriate birthweight for the child’s gestational age at birth, it is clear that Japan recommends stricter limiting of weight gain during pregnancy, compared to other developed countries^13^,^14^. In this context, pregnant Japanese women are expected to weigh themselves at every perinatal check-up and receive dedicated attention from their care provider during the entire pregnancy. Moreover, the majority of Japanese women believe that carrying a smaller baby would ensure a smooth delivery^15^, which can lead them to avoid extra weight gain during their pregnancy. However, a previous study found that women with weight gains <8 kg had a 2-fold higher risk of delivering a baby that was small for gestational age, compared to women with weight gains of 10.1–12.0 kg^15^. Therefore, it is possible that the increasing incidence of underweight Japanese women who are of reproductive age and their concern for weight gain during pregnancy may have contributed to the increasing prevalence of term LBW infants in Japan.

The present study also found that the risks of LBW and preterm birth were higher among women who were ≥35 years old, compared to younger women, although the adjusted odds ratio gradually decreased during the study period.

In this context, pregnancy at an older age has been considered a risk factor for adverse obstetric outcomes, such as LBW and preterm births^16^. In addition, the proportion of women who experience their first pregnancy after their mid-30s has gradually increased since the 1970s^7^. Previous studies have found that older mothers, and especially older primiparous mothers, are more likely to experience several obstetric complications, which can increase the risk of adverse outcomes for their infants. Moreover, older women are more likely to become pregnant after using ART because of their declining fertility, and several studies have found that the prevalences of preterm and LBW births are higher among women who used ART, compared to women who conceived naturally^17^,^18^,^19^. Although the proportion of ART-related births among older women has markedly increased during the study period, the risk of LBW and preterm births among older women has gradually approached that of younger women. In fact, Japanese vital statistic data showed a dramatic increase in the proportion of mothers over 35 years, from 4.2% in 1980 to 23.8% in 2010^3^. However, we found that the increase in prevalence of LBW infants among older mothers remained small, compared to that of younger mothers, and the gap in AOR for LBW infants has become smaller in recent years. This might be partially explained by the increasing risks among younger women. Despite the concern regarding obesity in most developed countries, Japanese population-based analyses have revealed a significant decrease in BMI and high incidences of low BMI, among 15–29-year-old women between 1979 and 2000^20^. Another study by the same group also found that younger women exhibited a greater desire to lose weight and greater concern regarding body image after childbirth^16^. Thus, the Health Japan 21 plan was released in 2000^21^, which aimed to promote health in the Japanese population. Its two specific goals were to reduce the prevalence of underweight 20–29-year-old women to <15% and to increase awareness regarding optimal weight by 2010. However, recent population-based data indicate that there have only been small changes in the prevalence of underweight young women from baseline^21^. Thus, increase in the risk of preterm and LBW births among younger women may be related to undernutrition and thinness, which may partially explain the decreasing odds ratios that we observed for these birth outcomes among older women.

The present study is limited by inadequate information regarding obstetric (e.g., using of ART), anthropometric, and socioeconomic characteristics of the parents and their infants. Furthermore, although we evaluated birth certificate data for all live births in Japan during the study period, we were only able to collect data regarding the place of birth, birthweight, maternal age, gestational age at birth, and parents’ occupation(s). Therefore, to accurately identify potential risk factors that explain the recent increases in LBW and preterm births, further studies must consider more detailed information that could affect obstetric outcomes, such as maternal BMI, weight gain during pregnancy, pregnancy complications, smoking status, and mode of delivery. Identification of these risk factors may facilitate the development of effective strategies to prevent further increases in LBW and preterm births in Japan.

In conclusion, the present study evaluated a Japanese dataset from 1979–2010 and identified trends of decreasing mean birthweight and increasing prevalence of LBW infants, especially for term LBW infants. We also found an increased risk of LBW infants, especially among women who were <35 years old. These trends may be related to changing patterns in young women’s anthropometric characteristics, lifestyles, and nutritional statuses. Additional studies are needed to identify the relevant risk factors, as their identification may help reduce the unnecessary long-term socioeconomic burden that is associated with LBW infants.

## Methods

Birth certificate data from January 1, 1979 to December 31, 2010 were obtained from the Ministry of Health, Labour and Welfare, which performs national population-based surveys each year^8^. This study was conducted in accordance with the principles of Declaration of Helsinki. Since government approval had been granted to use birth certificates with anonymous secondary data, approval by institutional review boards was not sought. We only considered data for singleton births to avoid the bias of a higher risk of LBW for multiple births, and several demographic variables were extracted for the analyses. Based on birthweight, every infant was categorized as LBW (a birthweight of <2,500 g) or normal birthweight (a birthweight of ≥2,500 g). Furthermore, every infant was also categorized based on gestational age as preterm (a gestational age of <37 weeks) or term (a gestational age of ≥37 weeks). Time trends were analysed using a linear regression model.

Logistic regression analyses were performed for each year to assess the association between maternal age and LBW/preterm births. According to maternal parity, adjusted odds ratios (AORs) for preterm birth and having a LBW infant were calculated as coefficient estimates for older mothers by using the following explanatory variables: infant sex, birthplace (urban area: 1, rural area: 0), and maternal age (≥35:1, <35:0). Birthplace is important for analysis, as maternal pre-pregnancy body mass index, total weight gain during pregnancy, duration of pregnancy, and rate of Caesarean section were shown to be significantly different between rural and urban areas in Japan^22^. Urban areas were defined based on their designation as a city or special ward, and all other areas were defined as rural areas. In addition, we assessed the AOR for maternal parity because older mothers, and especially older primiparous mothers, are more likely to experience several obstetric complications, which can increase the risk of preterm birth and having a LBW infant^9^. To evaluate the secular trend in the risk of delivery for older mothers, the estimated odds ratios and 95% confidence intervals (CIs) for maternal age were plotted using locally weighted polynomial regression as a smoothing technique. All database manipulations were performed by SQL and AWK using R software (version 3.1.2).

## Additional Information

**How to cite this article**: Takemoto, Y. *et al*. Japanese secular trends in birthweight and the prevalence of low birthweight infants during the last three decades: A population-based study. *Sci. Rep.* **6**, 31396; doi: 10.1038/srep31396 (2016).

## Figures and Tables

**Table 1:** Mean birthweight of singleton live birth and prevalences of preterm, low birthweight, and term low birthweight infants.

	1979 n = 1,638,371	1990 n = 1,215,492	2000 n = 1,184,171	2010 n = 1,068,051
Mean birthweight (g) ± SD				
Total*	3152 ± 436	3078 ± 429	3051 ± 420	3018 ± 421
Male*	3193 ± 442	3118 ± 435	3090 ± 427	3059 ± 427
Female*	3109 ± 426	3037 ± 418	3009 ± 409	2975 ± 409
Prevalence (%)				
Preterm	3.5	4.1	4.5	4.7
LBW	4.5	5.6	7.4	8.3
Term LBW	2.7	3.4	4.8	5.3

^*^p < 0.001.

**Table 2:** Prevalence of LBW infants according to maternal age.

Prevalence of LBW infants according to maternal age (%)				
	1979	1990	2000	2010
Age <25 years				
<1.0 (kg)	0.06	0.16	0.18	0.26
<1.5	0.29	0.48	0.49	0.58
<2.5	5.04	6.48	7.73	8.25
Age 25–34 years				
<1.0 (kg)	0.07	0.13	0.17	0.21
<1.5	0.28	0.39	0.45	0.50
<2.5	4.14	5.22	7.14	7.93
Age ≥35 years				
<1.0 (kg)	0.19	0.31	0.29	0.37
<1.5	0.76	0.84	0.82	0.90
<2.5	7.44	7.17	8.53	9.51

**Table 3:** Adjusted odds ratio of women ≥35 years compared to women <35 years.

Adjusted Odds Ratio of women ≥35 years (95% CI)				
	1979	1990	2000	2010
Preterm				
Primiparous	2.14 (2–2.28)	2.01 (1.91–2.12)	1.67 (1.60–1.74)	1.50 (1.46–1.55)
Multiparous	2.15 (2.07–2.22)	1.67 (1.62–1.73)	1.39 (1.35–1.43)	1.30 (1.26–1.33)
LBW				
Primiparous	2.06 (1.95–2.18)	1.69 (1.62–1.77)	1.48 (1.44–1.53)	1.34 (1.31–1.37)
Multiparous	1.90 (1.83–1.97)	1.42 (1.38–1.46)	1.20 (1.17–1.23)	1.21 (1.18–1.24)

Dependent variable: Preterm birth/LBW.

Explanatory variables: infant sex, birthplace (urban area: 1, rural area: 0), and maternal age (≥35 years: 1, <35 years: 0).
